# Long-Term Heavy Ketamine Use is Associated with Spatial Memory Impairment and Altered Hippocampal Activation

**DOI:** 10.3389/fpsyt.2014.00149

**Published:** 2014-12-04

**Authors:** Celia J. A. Morgan, Chris M. Dodds, Hannah Furby, Fiona Pepper, Johnson Fam, Tom P. Freeman, Emer Hughes, Christian Doeller, John King, Oliver Howes, James M. Stone

**Affiliations:** ^1^Centre for Clinical Psychopharmacology, University of Exeter, Exeter, UK; ^2^Clinical Psychopharmacology Unit UCL, University College London, London, UK; ^3^Neuroimaging Sciences, Cardiff University, Cardiff, UK; ^4^Institute of Psychiatry, Kings College London, London, UK; ^5^Department of Experimental Medicine, Imperial College London, London, UK; ^6^Donders Institute for Brain, Cognition and Behaviour, Nijmegen, Netherlands

**Keywords:** ketamine, drug abuse, hippocampus, memory, spatial memory, NMDA receptor

## Abstract

Ketamine, a non-competitive *N*-methyl-d-aspartate receptor antagonist, is rising in popularity as a drug of abuse. Preliminary evidence suggests that chronic, heavy ketamine use may have profound effects on spatial memory but the mechanism of these deficits is as yet unclear. This study aimed to examine the neural mechanism by which heavy ketamine use impairs spatial memory processing. In a sample of 11 frequent ketamine users and 15 poly-drug controls, matched for IQ, age, years in education. We used fMRI utilizing an ROI approach to examine the neural activity of three regions known to support successful navigation; the hippocampus, parahippocampal gyrus, and the caudate nucleus during a virtual reality task of spatial memory. Frequent ketamine users displayed spatial memory deficits, accompanied by and related to, reduced activation in both the right hippocampus and left parahippocampal gyrus during navigation from memory, and in the left caudate during memory updating, compared to controls. Ketamine users also exhibited schizotypal and dissociative symptoms that were related to hippocampal activation. Impairments in spatial memory observed in ketamine users are related to changes in medial temporal lobe activation. Disrupted medial temporal lobe function may be a consequence of chronic ketamine abuse and may relate to schizophrenia-like symptomatology observed in ketamine users.

## Introduction

The *N*-methyl-d-aspartate-receptor (NMDA-R) antagonist ketamine when given acutely has profound effects on many aspects of cognition and behavior. Subjective experiences of the drug include reports of unusual bodily sensations, such as shrinking or growing body parts, hallucinations, and “out-of-body” experiences. While these side effects are considered problematic when ketamine is employed as an anesthetic agent or, more recently, as a potential treatment for depression, the dissociative effects of ketamine have driven its popularity as a recreational drug worldwide. Ketamine is now one of the UK’s leading club drugs after marijuana, cocaine, and ecstasy (1) and is very popular in Hong Kong, where 87% of young drug users are estimated to be ketamine users (2). With prevalence rising worldwide in recent years (3), there is an urgent need to establish the consequences of long-term use of the drug on psychological wellbeing and neurocognitive function. Whilst an array of research has investigated the acute neurological and psychological consequences of ketamine administration [e.g., (4)], research addressing the long-term impact of the drug is sparse.

In a recent, large scale longitudinal study, we assessed cognitive function in a sample of frequent, infrequent, abstinent, and non-ketamine users and again 12 months later (5). In keeping with earlier reports (6), we found that ketamine-induced long-term cognitive deficits were confined almost exclusively to frequent users. Out of a wide battery of cognitive tests, heavy ketamine users exhibited numerous cognitive impairments, but particularly a specific decline in spatial working memory, which was crucially linked with increasing ketamine use over the year of testing. This suggests that this effect was mediated by ketamine use and not a result of pre-existing differences between ketamine users and controls. To date, only this study has investigated spatial memory following frequent ketamine use in humans, which is perhaps surprising given the extensive preclinical evidence of impairment of spatial memory with ketamine and other NMDA-antagonists [e.g., (7)] where findings indicate that hippocampal NMDA-receptor activity and NMDA-receptor-dependent plasticity are fundamental for spatial learning. In the only similar human study, Rowland et al. (8) found that spatial memory performance was significantly poorer than controls on a virtual MWM task following acute low-dose ketamine, whereas no impairments were seen in other cognitive tasks.

Spatial navigation to a hidden goal is thought to be supported by two different systems: a place or “allocentric” system driven by hippocampal-dependent learning of environmental layout which is viewpoint independent, and a taxon or “egocentric” system based on striatal-dependent learning of simple responses to individual stimuli from a person centered perspective (9). Findings from a variety of functional neuroimaging studies suggest that the hippocampus underpins accurate navigation to a target using “allocentric” memory whereas the dorsal striatum, in particular the caudate, is involved in action-based navigation from a learned response (“egocentric” memory) [e.g., Ref. (10)]. Both these systems are involved in successful navigation to a hidden goal and some evidence suggests that when one system is impaired the other may compensate. Evidence also suggests that the dorsal striatum (caudate) is also involved in updating spatial memories with new information (11).

Given the preliminary evidence of both acute and chronic ketamine-induced spatial memory deficits in human beings, along with the high density of NMDA receptors in the hippocampus and the pivotal role of the NMDA-receptor in learning, the present experiment aimed to explore spatial memory deficits observed in people who take ketamine frequently by investigating whether this impairment may be explained by differences in neural activity within the hippocampus. The task used in the current study was designed to be similar to the Morris Water Maze paradigm employed widely in rodent studies of hippocampal-dependent spatial memory and navigation. It was derived from that used in Ref. (11), involved the learning of several target locations in a virtual field, using corrective feedback over multiple trials. We hypothesized that chronic NMDA hypofunction in ketamine users would disrupt the construction of a rich contextual representation of the environment, thought to be necessary for the efficient navigation toward a learned goal. Therefore, ketamine users would be significantly poorer at navigating from memory compared to poly-drug matched controls, while navigation to a visible target would be similar across groups. We focused our analysis on three regions – the hippocampus, parahippocampal gyrus, and the caudate-predicting that heavy ketamine use might affect the recruitment of the hippocampus and parahippocampal gyrus to a greater degree than the caudate, given previous preclinical evidence of neurotoxicity in this region following chronic ketamine administration (12).

## Methods and Materials

### Participants

Eighteen ketamine users and 18 controls were recruited to the study through the database of existing drug users at UCL’s clinical psychopharmacology unit and via word of mouth. Ten subjects were excluded from the study for a variety of reasons. Five due to technical failure, three did not meet the ketamine usage criteria, one due to shivering in the scanner, and one pressed the panic button due to paranoia during scanning.

Complete data sets were collected for 11 ketamine users (8 male; 3 female) and 15 controls (10 male; 5 female) aged between 20 and 44 years old. Ketamine users were defined as those who had self-administered ketamine at least three times per week for the past year. Poly-drug using controls were broadly matched with the ketamine group according to: their use of alcohol and tobacco; age; gender; years in education; and premorbid IQ. Participants were required to abstain from psychotropic drugs for at least 24 h prior to testing, abstinence and recent drug use were verified with urinalysis. Participants were also required to have no history of head injury or psychiatric illness, to be right handed and fluent in English. The study was approved by both the Imperial College Research Ethics Committee and the UCL Graduate School ethics committee. All participants were paid for their participation and travel.

### Procedure

After giving informed consent, all participants were required to provide a comprehensive self-report of their drug use history. Urine samples were analyzed for verification of recent drug use. A personal and family history of mental illness, alcohol, and drug abuse was taken, and participants completed the Spot-the-Word test (13) as an index of verbal IQ. Participants then completed a training session in the virtual reality spatial memory task to be used in the scanner. Total scan time was approximately 90 min. Once the scans were finished, participants were guided to a quiet room where they completed assessments of psychopathology and neurocognitive function, detailed below.

### Spatial navigation task

#### Virtual reality environment

The task was adapted from that created by Doeller et al. (11) using UnrealEngine2 Runtime software (Epic Games). The virtual reality environment consisted of a first person perspective view of a grassy plain surrounded by distal mountains, clouds, and sun, projected at infinity to provide orientation but not location information. Surrounding the plain was a circular boundary, creating an arena in which there was positioned a single intramaze landmark (a traffic cone). The viewpoint was approximately two virtual meters above ground, with a field of view set at 90°. The arena was 180 virtual meters in diameter. The virtual heading and location was recorded every 100 ms.

#### Training

Participants were familiarized with the experimental task during training immediately prior to scanning. The training environment differed from the experimental environment to prevent any unspecific learning effects. These environments differed with respect to the shape of the environment (square), the ground (desert), the intramaze landmark (a tree and a bush), the global configuration of background cues, and the objects used. Participants learned the positions of four different objects and performed two trials per object. The experiment proceeded when the experimenter was satisfied that the task had been understood.

### Stimuli, task and trial structure

**Figure 1:**
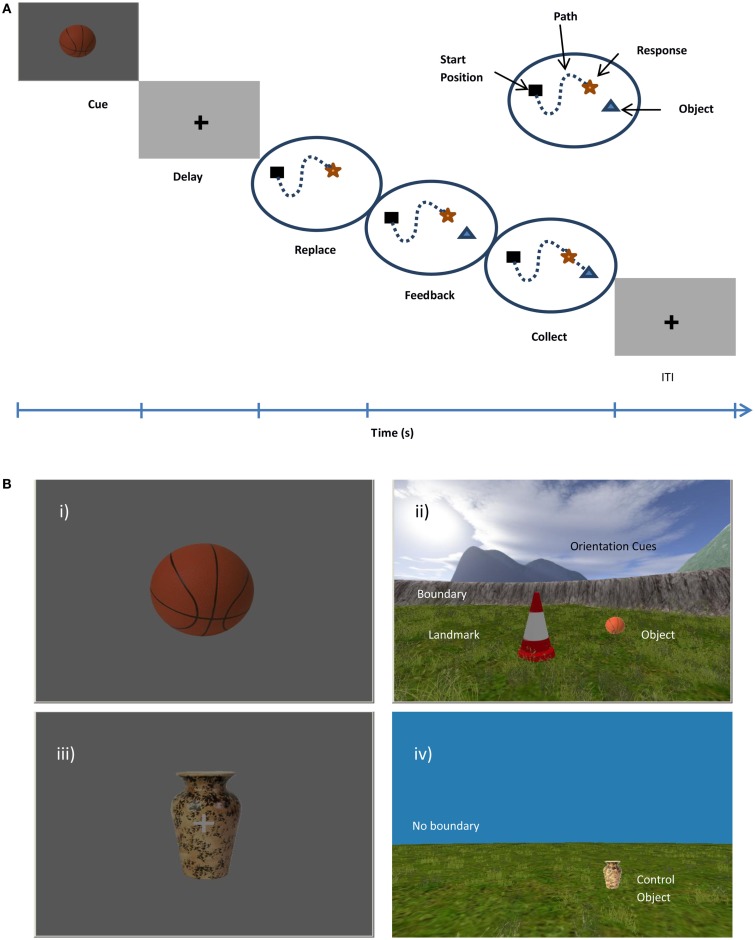
**(A)** Trial structure in experimental trials. Participants are presented with an object cue and after a short delay must replace the object in its remembered location. The object will appear in correct location and participant must walk over it to collect it. **(B)** Virtual Arena from a first person perspective. (i) In an experimental trial the object cue will appear on screen, e.g., ball. (ii) Once the object has been located and responded to, the object will appear in the correct location. On control trials (iii) the same object will be cued (i.e., vase) and (iv) the participant is required to walk directly to the visible location. Experimental trials contain an intramaze landmark (traffic cone), the boundary (circular wall), and orientation cues (mountains), control trials have no boundaries or landmark and object remains visible throughout trial.

Once the task had begun, participants were required to complete four initial learning trials to establish target locations for each object (See Figure 1). At the beginning of each learning trial a single cue (green man) appeared on the screen to signify for them to get an object that was somewhere in the environment. Participants looked for each of the four items individually, once only, on successive trials. On finding the object, participants were instructed to remember its location with reference to the position of the landmark, boundary and orientation cues. Walking over the object to collect it constituted the end of each trial.

During experimental trials (See Figure 1), participants were presented with a cue (one of the four objects) presented on a blank background for 2 s (cue phase). The cue signified participants to move, as accurately as possible, to the location in which that object was previously located, following a variable delay (fixation cross 2–6 s, mean 4 s). Using the right button keypad, participants navigated from a randomly varied start position to the remembered location and responded with the left index finger to indicate their response location (replace phase). The object then reappeared in it actual target location, the green man symbol appeared immediately if the response had been registered and signified for the participant to get the object. This allowed participants to re-encode the correct location of the object and update, using available cues before collecting it (feedback phase). Whereas the replace phase aimed to tap into memory retrieval, the feedback phase was thought to drive error-corrected learning by updating the spatial representation including relationships between the object and the boundary or landmark (See Figure 1A). In this sense, the participant continued to learn the exact location throughout each experimental block. A fixation cross was presented for a variable inter-trial interval (ITI, mean 6 s, jittered to avoid fMRI acquisition timing artifacts) before beginning the next trial.

Control trials were cued by a picture of an object, i.e., a vase (Figure 1Aiii), which remained the same across every control trial. After a short delay, the participant had to collect the visible object from an infinite grassy plane with no boundary, intramaze landmark, or orientation cues (Figure 1Aiv). In this way, these trials could be directly compared to the feedback phase of the experimental cues which required a route-following navigation strategy without requiring spatial memory or spatial processing of the environmental location. Cue, delay, and ITI durations were the same as the experimental trials.

#### Design

Overall there were four experimental trials per object, totaling 16 trials per block presented in pseudorandom order. There were three experimental blocks and control trials were interspersed throughout. The location of the landmark was moved each block.

### fMRI acquisition

All imaging took place in a 3-T Phillips intera magnetic resonance scanner (Medical Systems, Best, The Netherlands) equipped with a SENSE head volume coil located at the Robert Steiner Magnetic Imaging Center at The Hammersmith Hospital. Functional BOLD-sensitive T2* weighted functional images were acquired using a single shot gradient-echo EPI pulse with the following parameters: TR = 3000 ms, TE = 30 ms, flip angle 90°, slice thickness = 3.25 mm, slices = 44, slice gap = 0 mm, in-plane resolution = 2.19 mm × 2.19 mm, 32 slices per volume parallel to the AC-PC line. The first five volumes were discarded as dummy scans to allow for T1 equilibration. Participants were began by carrying out a short 2 min resting state scan, followed by the virtual reality spatial memory task during fMRI. The duration of the virtual reality task was dependent on the speed of participant performance. On completion, participants underwent a T1* structural scan followed by a 1H-Magnetic Resonance Spectroscopy (MRS) scan. Data from the resting state fMRI and MRS scan are reported elsewhere (14).

### fMRI analysis

Using SPM8 (Wellcome Department of Cognitive Neurology), images were spatially realigned to the first image in the time series, visually inspected for evidence of artifacts, and corrected for distortions according to the field map. Images were normalized to the standard Montreal Neurological Institute reference brain and spatially smoothed with an isotropic FWHM Gaussian kernel of 6 mm.

fMRI time series was modeled by a general linear model (GLM) including regressors for cue, replace, and feedback phases for each type of trial (memory or control). In the first level analysis, all regressors were convolved with the SPM hemodynamic response function. Data were high-pass filtered (cutoff period 1/120 HZ) and coefficients for each regressor were estimated using a least-mean-squares fit of the model to the time series for each participant.

The virtual reality task adopted by this study was chosen in order to assess the neural correlates associated with spatial learning and the different strategies used to navigate to a learned location.

A first level analysis computed linear contrasts of coefficients for each participant specified as:

1. Navigation from cue offset to the point of dropping the object compared to navigation to visible object in control trials. This contrast revealed regions which are active when navigating from memory.
2. Activation from when object was shown in correct location and its subsequent collection (updating of spatial memory) compared to walking to a visible object in control trials.
3. Walking toward a visible object compared to rest.

Contrast images from these first level contrasts were taken to second level group analyses treating subject as a random effect. Independent sample *t*-tests were used to test for effects at the group level.

### Regions of interest

**Figure 2:**
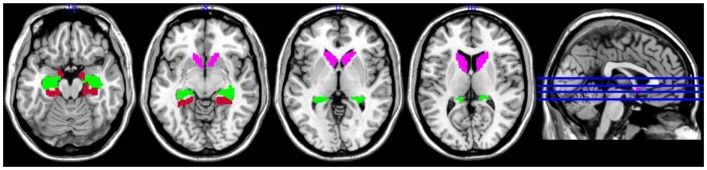
**Regions of interest superimposed on MNI single subject template brain; pink = caudate, green = hippocampus, red = parahippocampal gyrus**.

We restricted our analyses to those brain regions found to play key roles in navigation and spatial updating (See Figure 2): the hippocampus, parahippocampal gyrus, and caudate nucleus (11). Regions of interest were defined anatomically using the AAL atlas. Left and right ROIs were analyzed separately. ROI analyses were implemented using the Marsbar toolbox (15) and corrected for multiple comparisons. All *p*-values reported are corrected.

### Neurocognitive assessments

**Spot-the-Word** (13) was used as an index of pre-morbid IQ.
**Prose recall subtest of the Rivermead Behavioral Memory Test** (16) tapped episodic memory. Participants were required to recall verbally as much as they could remember of a passage of prose both immediately and after a delay.
**Spatial N-back** tapped spatial working memory. This is a computer based task in which visual stimuli appeared sequentially in one of six different locations around the center of the screen. Participants were required to give “Yes” or “No” button responses according to whether the stimulus was presented in the same position as the stimulus (1) one before (1-back), and subsequently, (2) two before (2-back). The N-back was analyzed using the signal detection theory (17). D prime (*d*′) scores were calculated as a sensitive measure of accuracy using the standardized difference between the hit rate (signal) and false alarms rate (signal + noise) using the equation: *d*′ = z(H) − z(F). *C* scores were also calculated as an indicator of response bias, i.e., the inclination to say no using the equation *C* = 0.5[z(H) + z(F)].
**Trailmaking Test** (18) indexed psychomotor speed (part A) and executive functioning (B–A).
**Verbal and Semantic fluency** tasks were used to tap into executive functioning and retrieval from semantic memory. Participants were provided with a single letter, e.g., “V” or a superordinate category, e.g., four-footed animals, and asked to generate as many exemplars as possible within 60 s.

### Psychological well-being

Questionnaires used to tap psychological well-being were: **Dissociative Experiences Scale** [DES (19)]; **Beck Depression Inventory** [BDI; (20)], which assessed depressive symptoms over the previous week; **State-trait anxiety inventory** [STAI; (21)]; **Peter’s Delusion Inventory** [PDI; (22)] assessed delusional symptoms; **Schizotypal Personality Questionnaire** [SPQ; (23)] assessed schizotypal symptoms using DSM-III-R criteria for schizotypal personality disorder; **Leeds Dependence Questionnaire** [LDQ; (24)] measured dependence upon ketamine.

### Analysis of neurocognitive measures and correlations

Data were analyzed using SPSS Statistics 19 (IBM). Data were checked for assumptions of normality and heterogeneity of variance and then subjected to *t*-tests or repeated-measures ANOVAs where appropriate. Correlations calculated Pearson’s correlation coefficient where data were parametric, corrected for multiple comparisons using the Bonferroni correction.

## Results

### Demographics

A total of 26 participants completed the study: 11 frequent ketamine users and 15 poly-drug-matched controls. Independent sample *t*-tests revealed that the groups were demographically well-matched, with no differences in age, number of years spent in education or premorbid IQ as indexed by the “Spot-the-word” test across the two groups (see Table 1). Although a higher proportion of males than females were included in the study, there were no differences in gender balance at the group level. There were also no differences in the numbers of individuals with a personal mental illness, family mental illness, family alcoholism, or family drug abuse.

**Table 1:** **Demographic data (mean ± SD) for ketamine users and controls**.

	Ketamine users	Poly-drug controls
No. of subjects	11	15
Age	28 ± 4.03	26.13 ± 5.25
Gender (male/female)	8/3	10/5
Years in education	13.80 + /3.29	16.47 ± 3.96
Spot-the-word	47.4 ± 7.09	46.93 ± 5.85
Personal history of mental illness	1/11	1/15
Family history of drugs	2/11	1/15
Family history of mental illness	4/11	3/15
Family history of alcoholism	2/11	1/15

### Ketamine use

All 11 participants in the ketamine group reported taking ketamine at least three times per week for at least 1 year, with last use occurring an average 1.6 (±0.70) days prior to testing (See Table 2). Participants in the ketamine group reported taking ketamine for a mean of 9.7 ± 3.62 years, a mean of 5.0 ± 1.15 days per week. Subjects reported taking a mean of 3.9 ± 3.73 g per session and 10.2 g (±7.42) per week. Ketamine users scored more highly than controls on the Leeds Dependence Questionnaire [*t*(24) = 7.59, *p* < 0.001].

**Table 2:** **Group means and SD for subjective report of drug use habits**.

		Ketamine users	Poly-drug controls
Cannabis	No. of regular users	8/11*	4/15
	Amount used (time to smoke 1/8 oz)	2.56 ± 2.19	15.00 ± 12.66
	Frequency (days/month)	21.00 ± 12.25*	15.5 ± 12.77
	Last used (days)	3.00 ± 4.87	4.50 ± 6.35
	Years of use	13.88 ± 3.44*	8.00 ± 2.45
Ecstasy	No. of regular users	2/11	0/15
	Amount used (mg/session)	277.78 ± 253.86	
	Frequency (days/month)	1.18 ± 1.49	
	Last used (days)	219.75 ± 305.79	
	Years of use	10.22 ± 6.28	
Cocaine	No. of regular users	4/11*	1/15
	Amount used (g/session)	2.13 ± 1.44	1.44
	Frequency (days//month)	6.50 ± 3.70	2.00
	Last used (days)	6.75 ± 9.54	3.00
	Years of use	8.75 ± 1.26	7.00
Alcohol	No. of regular users	9/11	15/15
	Amount used (units/session)	6.89 ± 5.56	5.00 ± 3.38
	Frequency (days//month)	15.33 ± 9.95*	7.00 ± 5.45
	Last used (days)	2.22 ± 2.99	3.60 ± 3.64
	Years of use	15.56 ± 3.43	10.80 ± 6.60
Tobacco	No. of regular users	10/10	13/15
	Amount used (cigarettes/session)	10.30 ± 5.90*	3.31 ± 5.51
	Frequency (days//month)	24.3 ± 10.94*	11.31 ± 14.06
	Last used (days)	0.60 ± 0.70	1.5 ± 4.37
	Years of use	12.90 ± 6.24*	4.92 ± 8.40

*p = 0.05.

### Other drug use

Groups were matched for the number of regular tobacco and alcohol users, however, there were significantly more regular cannabis and cocaine users in the ketamine group. Two out of the 11 ketamine users rated themselves as regular ecstasy users whilst none of the control group met the criteria as regular users. Regular users were defined as those who had used the drug once or more within the preceding 30 days. The ketamine group reported smoking both tobacco and cannabis more frequently and for more years than the control group. While the ketamine group reported smoking a larger quantity of cigarettes per day than controls, the days taken to smoke “an eighth” (3.5 g) of cannabis (an index of amount used) did not differ across groups. Groups were matched for the duration, frequency, and amount of cocaine used. Groups were also matched for the duration and quantity of alcohol consumed per session, although the ketamine users reported drinking alcohol more frequently (Table 2).

### Spatial memory task

#### Behavioral data (Figure 3)

Behavioral performance on the virtual reality task data was collected in the form of “virtual units” of distance error between the replaced object location and the correct object location. Data was collected for every participant on each trial within the three blocks. A large number of time-out trials meant that we were unable to analyze performance on a trial-by-trial basis, instead we calculated a mean score for each block. Control trials were not included in the analysis as there was no behavioral measure on these trials.

**Figure 3:**
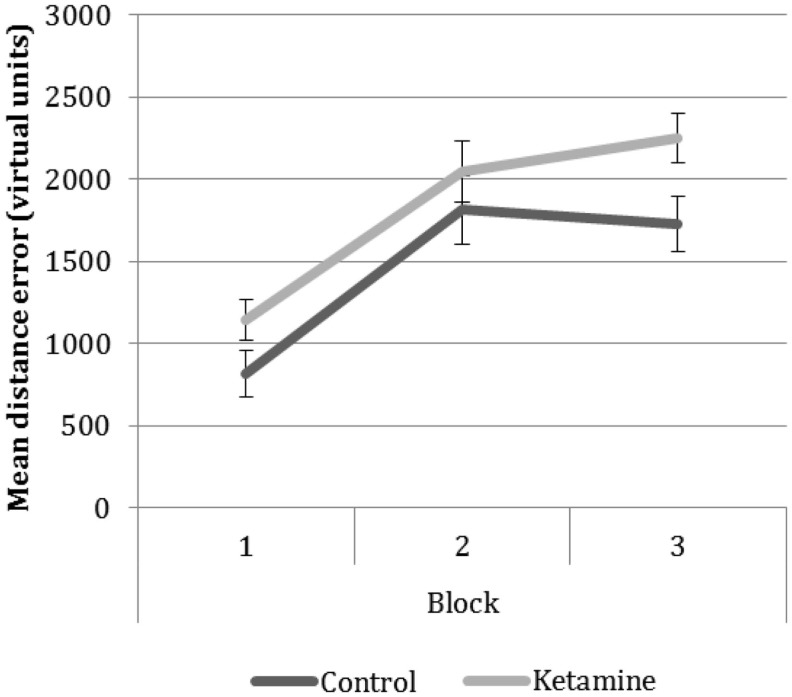
**Group performance on the ‘replace’ phase of the task over the three blocks**. Scores reflect the distance error between the location the participant placed the object in and its correct location.

Navigational performance was in a 2 × 3 ANOVA (group × block). There was a main effect of group *F*_1,23_ = 4.42, *p* = 0.04, attributable to greater error in the ketamine users across all blocks, and a main effect of block (*F*_2,56_ = 33.72, *p* < 0.001). Pairwise comparisons revealed significant differences in performance between block 1 and the subsequent blocks reflecting poorer memory performance in the later blocks compared to the first block. No significant difference was seen between mean performances on the two later blocks.

### fMRI data

#### Contrast 1: Navigation from memory

**Figure 4:**
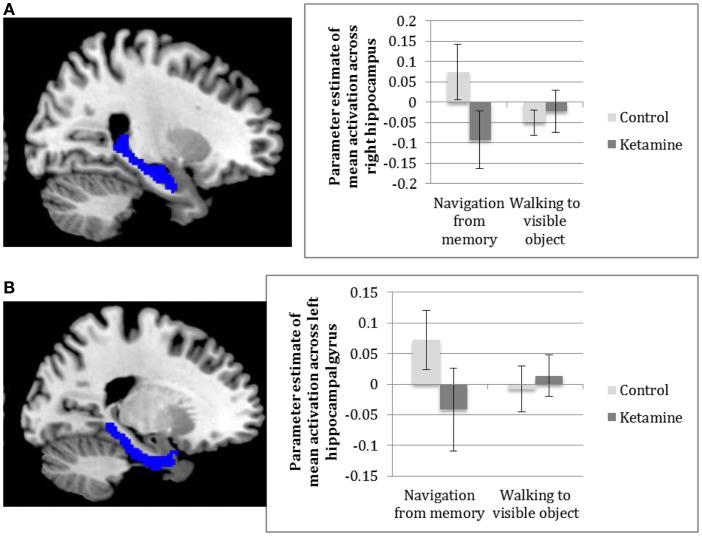
**Parameter estimates across (A) the right hippocampus (B) left parahippocampal gyrus in ketamine users and controls for navigating from memory compared to walking to a visible object**.

Analysis of the individual ROIs between groups revealed significantly greater activation of the right hippocampus [*t*(24) = 2.77, *p* = 0.03], as shown in Figure 4A, and the left parahippocampal gyrus [*t*(24) = 2.81, *p* = 0.03], Figure 4B, in the control group compared to the ketamine group for navigation from memory compared to walking to a visible object, with similar findings approaching significance in the right parahippocampal gyrus [*t*(24) = 2.42, *p* = 0.07] and left caudate [*t*(24) = 2.35, *p* = 0.08] (See Figure 4). Examination of the main effect of task across both groups found no significant mean activation across regions during navigation from memory relative to navigation toward a visible cue. Analysis of individual within-group activation revealed highly significant activation of the right hippocampus [*t*(14) = 3.74, *p* = 0.003] and right parahippocampal gyrus [*t*(14) = 3.51, *p* = 0.006] and significant activation of the left hippocampus [*t*(14) = 3.21, *p* = 0.021]and left parahippocampal gyrus [*t*(14) = 3.14, *p* = 0.014] in controls, again for the main effect of task. None of these regions showed significant activation in the ketamine group.

### Spatial memory updating from feedback

**Figure 5:**
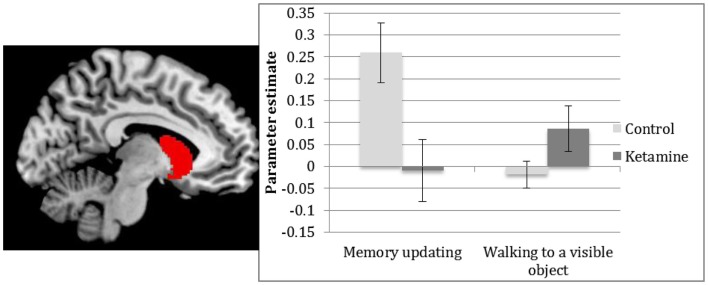
**Parameter estimates of activations in the left caudate for ketamine users and controls between receiving feedback on the correct location of object and collecting the object (memory updating) and walking to a visible object**.

ROI analysis revealed greater activation in controls compared to ketamine users in the left caudate [*t*(24) = 2.6, *p* = 0.048] when updating spatial memory with feedback compared to control (walking to a visible object), see Figure 5. There were no significant main effects of task.

### Navigating to a visible object

Analysis showed no group differences or a main effect of task in activation within our regions of interest during navigation to the visible control object compared to simple cue processing.

### Cognitive assessments

#### Trailmaking

Groups differed significantly on the Trailmaking A task of psychomotor speed [*t*(24) = 2.79, *p* < 0.05], with slower response in the ketamine group [*t*(24) = 2.79, *p* < 0.05], however, groups did not differ on their raw scores on Trailmaking B task of executive function [*t*(24) = 1.13, *p* = 0.27] (See Table 3). Group scores of cognitive flexibility, a measure of executive function after accounting for differences in psychomotor speed (Trailmaking B – Trailmaking A), were not significantly different [*t*(13.48) = −0.48, *p* = 0.64].

**Table 3:** **Group scores on neurocognitive assessments (Mean ± SD)**.

	Ketamine users	Poly-drug controls
Trail making A	33.80 ± 10.48*	24.33 ± 6.59
Trail making B	46.20 ± 14.53	40.07 ± 12.41
Trail making total (B–A)	12.40 ± 19.81	15.73 ± 12.07
Verbal fluency	7.50 ± 3.21	8.53 ± 2.70
Category fluency	17.70 ± 4.52	17.33 ± 3.29
Prose recall immediate	6.45 ± 2.42	7.87 ± 3.43
Prose recall delayed	5.80 ± 2.16	7.50 ± 3.15
N-back (1-back)
No. correct responses	38.20 ± 11.30	43.62 ± 10.23
No. false alarms	7.50 ± 6.24	3.15 ± 3.96
d prime score	1.98 ± 1.13*	3.05 ± 1.19
C score	0.10 ± 0.22	0.10 ± 0.22
N-back (2-back)
No. correct responses	27.00 ± 12.74	35.62 ± 13.12
No. false alarms	12.60 ± 6.50	7.77 ± 6.26
*d* prime score	0.93 ± 1.26*	1.85 ± 1.28
*C* score	0.23 ± 0.28	0.13 ± 0.31

*p < 0.05; **p < 0.01.

#### Fluency

The number of words generated did not differ significantly between groups on verbal [*t*(24) = −0.87, *p* = 0.39] or category fluency [*t*(24) = 0.24, *p* = 0.86].

#### Prose recall

A repeated-measures ANOVA showed a significant main effect of delay (*F*_1,23_ = 8.77, *p* < 0.007] in which delayed recall was significantly poorer than immediate recall across both groups, the main effect of group was not significant [*F*_1,23_ = 1.72, *p* = 0.2].

#### Spatial N-back

Due to computer error, data for two participants in the control group could not be collected and is therefore not included in the analysis. Using a repeated-measures ANOVA, a significant main effect was found between the *d*′ scores on the low and high load tasks [*F*_1,22_ = 19.06, *p* < 0.001] reflecting greater difficulty in manipulating spatial information than maintaining it. There was also a main effect of group (*F*_1,22_ = 4.99, *p* < 0.05) whereby accuracy was higher in the control than the ketamine group (see Table 3.) The interaction between load and group was not significant (*F*_1,21_ = 0.08, *p* = 0.79). A further repeated-measures ANOVA was carried out to assess for response bias between groups on the low and high load tasks. *C* scores were not significantly different between load (*F*_1,21_ = 2.31, *p* = 0.14) or group (*F*_1,21_ = 0.27, *p* = 0.61).

### Psychological wellbeing

Significant group differences were found on all three factors underlying schizotypal traits (see Table 4). The ketamine group scored higher on the Cognitive/Perceptual [*t*(22) = 2.70, *p* < 0.05], Disorganization [*t*(24) = 3.55, *p* < 0.005] and Interpersonal [*t*(22) = 2.17, *p* < 0.05] factors. There were significantly greater depressive symptoms on the BDI [*t*(23) = 2.44, *p* < 0.05] and anxiety on the STAI [*t*(24) = 3.82, *p* < 0.001] in the ketamine users compared to controls. The ketamine group scored more highly on two scales of the Peters Delusion Inventory. The distress [*t*(24) = 2.27, *p* < 0.05] and conviction [*t*(24) = 2.49, *p* < 0.05] subscales reached significance, while Preoccupation scores did not differ between groups [*t*(23) = 1.82, *p* = 0.08]. Significant differences were seen between the two groups on the Dissociative Experience Scale, in which the ketamine group scored more highly than controls [*t*(24) = 2.54, *p* < 0.05].

**Table 4:** **Group scores on assessments of psychological wellbeing (Mean ± SD)**.

	Ketamine users	Poly-drug controls
SPQ cognitive/perceptual	9.60 ± 8.54*	3.00 ± 2.88
SPQ interpersonal	9.56 ± 8.16*	4.47 ± 3.23
SPQ disorganization	9.20 ± 3.91*	3.07 ± 4.43
BDI	15.40 ± 9.18*	6.27 ± 9.16
STAI	44.60 ± 8.92**	33.20 ± 6.04
PDI distress	12.90 ± 12.02*	3.67 ± 5.52
PDI conviction	19.50 ± 19.50*	5.40 ± 8.46
PDI preoccupation	14.60 ± 19.82	4.47 ± 7.21
DES	27 ± 21.67*	8.95 ± 7.67
LDQ	15.25 ± 5.65**	1.13 ± 3.34

*p < 0.05; **p = 0.01.

#### Correlations

Parameter estimates were extracted from the first level analysis for right hippocampal activation and were correlated with error in spatial memory performance on the virtual reality task, schizotypal, and dissociative symptoms and amount of ketamine taken per day in the ketamine group only to limit the chance of Type I error. There was a negative correlation between mean error and hippocampal activation for navigation trials in the ketamine group (*r* = −0.78 *p* = 0.008). There were also highly significant negative correlations between total SPQ score (*r* = −0.816, *p* < 000.1) and total DES score (*r* = −0.861, *p* < 0.001) and hippocampal activation for navigation trials. A negative correlation between amount of grams of ketamine taken per day and hippocampal activation approached significance following Bonferroni correction (*r* = −0.696, *p* = 0.025).

## Discussion

The main findings of this study were that ketamine users showed significantly less activation within the hippocampal complex (including the right hippocampus and left parahippocampal gyrus) than controls, when navigating to a learned object location in a spatial memory task. Activation within the left caudate was also significantly greater in the control group than the ketamine group during memory updating. There were no group differences in activation when moving toward a visible object, tentatively suggesting a specific effect of frequent ketamine use on spatial memory.

Given the pivotal role of the hippocampal complex in spatial memory, it is compelling that we see such strong differences in neural activity between ketamine users and controls in the right hippocampus and parahippocampal gyrus during retrieval from spatial memory. Our results provide the first human data to suggest a long-term neural impact of frequent ketamine on spatial memory processes. Both the hippocampus and the caudate have been implicated in successful navigation to a target object, with the former thought to be responsible for retrieval of a cognitive map and the latter thought to drive action-outcome learning that enables successful navigation to a target over time (11). In light of our present findings, we can conclude that the functional activation of these regions is disrupted in ketamine users. Behavioral data also demonstrated spatial memory deficits in ketamine users, as we hypothesized, and convincingly these deficits were negatively correlated with hippocampal activation in ketamine users.

Given previous findings of the relationship between ketamine use and spatial memory deficits (25), and a correlation between degree of ketamine use and right hippocampal activation observed in this study, the reduced activation observed in both the hippocampal and caudal regions in our ketamine group are likely due to repeated use of this drug. Whether the disruption in activation on a spatial memory task observed in this study is due to structural damage in the hippocampus is unclear. The hippocampus contains two major cell types, glutamatergic pyramidal cells and GABAergic interneurons. GABA containing interneurons are 10 times more sensitive to NMDA-receptor antagonists than the pyramidal neurons (26), of which parvalubumin (PV+) interneurons are thought to be most affected by the drug. It is possible that chronic ketamine inhibits PV+ interneurons thereby disinhibiting excitatory pyramidal cells in the hippocampus, which in turn leads to widespread glutamate-mediated excitotoxicity through AMPA and kainite receptors (27). A significantly reduced density of PV+ interneurons has been found in the granule layer of the dentate gyrus and the CA1-CA3 pyramidal cell layers following repeated doses of ketamine in rats (28) and mice (29). “Place” cells are hippocampal pyramidal cells and are thought to be responsible for the encoding, construction and retrieval of a cognitive map of the environment, necessary for efficient navigation (9). A reduction of PV+ cells within this region could give rise to the reduced activation we see in our ketamine group during navigation from memory and consequent spatial memory impairment.

Navigation from memory also produced greater activation in the parahippocampal gyrus bilaterally in the control group but not the ketamine group. The parahippocampal gyrus is thought to encode the distance and allocentric direction of landmarks (e.g., large objects or barriers) in the environment, within which neurons are thought to be broadly tuned to a those located at a specific bearing from the navigator. Bidirectional associations between the parahippocampal gyrus and perirhinal areas (thought to integrate landmark locations with visual features), as well as between the parahippocampal gyrus and hippocampus, place the parahippocampal gyrus in an ideal position to drive the hippocampus (10) in a way that pattern completion of the firing rates of place cells in the dentate gyrus are always consistent with the navigator being in a single location (30). Our results are in line with these previous findings, which highlight the importance of the parahippocampal gyrus in supporting a representation in spatial memory.

Differences between ketamine users and controls were observed in the right, but not left hippocampus. The right hemisphere has been well implicated in studies of spatial navigation, in which it is thought to predict the use of an allocentric spatial representation whereas the left hippocampus is involved in the sequential organization of successive choices within such an episode that may be combined to support different aspects of navigation and episodic memory (31). In our study activation in controls was observed in both the left and right hippocampus but it may be that the weaker task-related activation of the left is the reason for us only observing differences on the right.

A reduction in the activation of the caudate nucleus during memory updating was also observed in ketamine users compared to controls. NMDA-receptor blockade is thought to disrupt learning in the striatum and prevents instrumental control in the form of learned action-outcome contingencies (A-O) or “goal-directed” actions (32). The caudate has been found to express long-term potentiation (LTP) that depends on the activation of both dopamine receptors and NMDA glutamate receptors. In our task, memory updating is assumed to occur in the time between replacing the object from memory and collecting the object from the correct location following feedback. This process of updating presumably invokes a prediction error signaling mechanism, in which the navigator is required to learn from the error distance and adjust their spatial memory accordingly. Given studies that have found glutamate-mediated dopamine depletion in the brain following chronic ketamine use (33), disruptions in prediction error signaling following acute ketamine (34) and given the role of intact dopaminergic function in the coding of prediction errors in the midbrain (35), it is possible that reduced caudal activation in our ketamine sample is a result of NMDA hypofunction, which impairs dopaminergic error signaling in the associative cortico-basal ganglia system, including the caudate and hippocampus. Glutamate modulates dopamine signaling at the level of glutamatergic afferents to the PFC, which in turn modulate concentrations of dopamine in the striatum via excitatory inputs to GABA neurons. We therefore speculate that the reduced activation of the caudate observed in ketamine users during memory updating is a result of a series of downstream modification of dopamine receptors related to NMDA hypofunction. Indeed, this is supported by recent preclinical work that suggests that spatial memory deficits in rats are driven by striatal dopaminergic changes (36).

Spatial working memory deficits were observed behaviorally on the N-back task, replicating previous findings (5) and we extended these findings by demonstrating an impairment on a virtual reality spatial memory task. In line with the findings from Doeller et al. (11) performance on Block 1 was significantly better than Block 2 and 3. This was due to the landmark moving in each block, making the task more difficult as previous locations interfere with memory. Extraction of parameter estimates allowed us to compare behavioral performance with neural activity at different stages within the task. A negative correlation between hippocampal activity and error in the ketamine group supports our hypothesis, in which hippocampal activity is thought to underpin spatial memory performance. Contrary to the findings of previous studies measuring the impact of chronic ketamine administration (5, 37), the ketamine group performed as well as controls on all other cognitive tasks, with exception of the spatial memory tasks in which ketamine users performed significantly worse than controls. This may in part be due to better educational and IQ matching of our sample with the control group than in previous studies. In accordance with numerous previous studies into the psychiatric consequences of ketamine use, subjects in this study scored greater than controls on measures of depression, dissociative experiences, anxiety, schizotypy, dependency and delusional experiences [see Morgan et al. (38) for a review]. This provides support for evidence demonstrating the sustained dissociation and schizophrenic-like symptoms in frequent ketamine users when drug free (39).

Intriguingly, schizotypal and dissociative symptoms were negatively correlated with hippocampal activation in ketamine users. This may be partly mediated by the negative correlation with degree of ketamine use found in the ketamine users, i.e., that heavier ketamine use results in both hippocampal dysfunction and greater schizotypal and dissociative symptoms. However, the correlations with symptoms were of a greater magnitude than this correlation, so this may relate to the putative role of the hippocampus in the genesis of schizophrenia. An inability to recruit the hippocampus during memory tasks in patients with schizophrenia has been repeatedly observed [e.g., Ref. (40)] and has been proposed to have a core etiological role in the disorder (41). Increasing hippocampal dysfunction in humans has been found to be associated with the transition from the prodromal psychotic state to acute psychosis (29) and the latter study demonstrated that a similar pattern of hippocampal dysfunction could be invoked following repeated ketamine administration in a mouse model. It has been demonstrated that a decrease in GABAergic function within the hippocampus, of the kind that is posited to happen following chronic ketamine administration, can also lead to increases in dopaminergic activation in the ventral tegmental area of rats (41). This may be one way in which the hippocampal disruptions observed in ketamine users in this study produce increases in schizophrenia-like symptoms, an explanation, which also fits with theoretical accounts of the link between repeated NDMA-antagonist use and schizophrenia (42, 43) that propose dopaminergic sensitization following chronic ketamine use. A recent theory has also suggested that glutamatergic disruptions in hippocampal synaptic plasticity may directly impact on symptoms by impairing the efficiency of hippocampal pattern separation, resulting in spurious associations of the nature observed in delusions (44). Future work with this population of users could aim to address more directly the links between glutamatergically mediated neurological changes and symptomatology in this group of individuals.

Methodological considerations common to most recreational drug research also apply to our study. All ketamine users were poly-drug users and the nature of the group meant that matching them with controls for other drug use was difficult, which limits how far we can attribute the effects seen in the experimental group exclusively to the effects of ketamine. There may also be pre-existing differences between groups that draw users to take ketamine (45), hence, it is hard to attribute the effects to chronic ketamine use *per se*. In this study, we attempted to overcome some types of pre-existing differences by matching groups for premorbid IQ, years in education, personal mental illness, family history of mental illness. For a study of this kind, groups were relatively well-matched for drug use with the exception of cocaine and MDMA cannabis, where there were more regular users but for alcohol and tobacco use the ketamine group also showed indications of higher use on some self-reported metrics. It is also important to stress that the recruitment criteria (absence of serious psychiatric comorbidity and high literacy) used in this study, may have resulted in a sample of highly functioning ketamine users, which might not be representative of the majority. It is also important to stress that the recruitment criteria (absence of serious psychiatric comorbidity and high literacy) used in this study, may have resulted in a sample of highly functioning ketamine users, which might not be representative of the majority.

In summary, this study of a sample of chronic ketamine users found impaired spatial memory and associated reduced activation in three key regions of the brain known to be involved in successful spatial navigation, the hippocampus, parahippocampal gyrus and the caudate nucleus. Hippocampal changes were also related to schizophrenia-like symptoms observed in ketamine users. Whilst we do not yet know how long-lasting these neural changes are, cautionary advice should be provided to the growing population of recreational users of the damaging effects of ketamine on the brain and aspects of cognitive function.

## Conflict of Interest Statement

The authors declare that the research was conducted in the absence of any commercial or financial relationships that could be construed as a potential conflict of interest.
